# PRIVACY AND SECURITY FOR TELEHEALTH DEVICES

**DOI:** 10.1093/geroni/igz038.3081

**Published:** 2019-11-08

**Authors:** London Thompson, Csilla Farkas

**Affiliations:** 1 Claflin University, Columbia, South Carolina, United States; 2 University of South Carolina, Columbia, South Carolina, United States

## Abstract

In this research, we study the privacy and security capabilities provided by telehealth devices. Our aim is to evaluate how vulnerable these popular devices are in the presence of malicious cyber attackers. As older adults increasingly rely on telehealth devices, it is crucial that cybersecurity aspects of these devices are clearly communicated to them. Moreover, older adults frequently lack the technical expertise to evaluate the security and privacy capabilities of the devices. The lack of control over telehealth devices is a major concern for older adults. Older adults view certain limitations within these devices as decreasing their privacy and security. These limitations include the lack of control over accepting calls, taking screenshots, and assigning access privileges. For large scale adaptation of telehealth devices by older adults, it is crucial that these devices not only satisfy their intended purpose but also exhibit user friendly features and strong security and privacy capabilities. Modeling cyber threats against telehealth devices is not studied sufficiently . Malicious actors may compromise telehealth devices and create further threats to security and privacy of the users. In this research, we studied the cyber threats against telehealth devices. We built a threat model that ranks cyber threats based on their impact. We investigated how the operating system of popular devices supports access control. We found that none of the current technologies support location-based access control. We claim that this represents a major limitation and that supporting location-based access control is necessary to ensure users’ privacy in their own home.

